# Structural Insights into Isovaleryl-Coenzyme A Dehydrogenase: Mechanisms of Substrate Specificity and Implications of Isovaleric Acidemia-Associated Mutations

**DOI:** 10.34133/research.0661

**Published:** 2025-05-28

**Authors:** Kaide Ju, Fang Bai, Youwei Xu, Qingao Li, Gengchen Su, Ye Jin, Houzao Chen, Shuyang Zhang, Xiaodong Luan

**Affiliations:** ^1^ School of Basic Medical Sciences, Tsinghua University, Beijing 100084, China.; ^2^ Department of Cardiology, Peking Union Medical College Hospital, Peking Union Medical College and Chinese Academy of Medical Sciences, Beijing 100730, China.; ^3^ The CAS Key Laboratory of Receptor Research, Shanghai Institute of Materia Medica, Chinese Academy of Sciences, Shanghai 201203, China.; ^4^ Department of Rare Diseases, Peking Union Medical College Hospital, Peking Union Medical College and Chinese Academy of Medical Science, Beijing 100730, China.; ^5^ Department of Biochemistry and Molecular Biology, State Key Laboratory of Medical Molecular Biology, Institute of Basic Medical Sciences, Peking Union Medical College and Chinese Academy of Medical Sciences, Beijing 100730, China.; ^6^ Tsinghua-Peking Center for Life Sciences, Tsinghua University, Beijing 100084, China.; ^7^ Center for Drug Research and Evaluation, Institute of Clinical Medicine, Peking Union Medical College Hospital, Beijing 100730, China.

## Abstract

Isovaleryl-CoA (coenzyme A) dehydrogenase (IVD) plays a pivotal role in the catabolism of leucine, converting isovaleryl-CoA to 3-methylcrotonyl-CoA. Dysfunction of IVD is linked to isovaleric acidemia (IVA), a rare metabolic disorder characterized by the accumulation of toxic metabolites. In this study, we present the cryo-electron microscopy structures of human IVD, resolved both in its apo form and in complex with its substrates, isovaleryl-CoA and butyryl-CoA. Our findings reveal a tetrameric architecture with distinct substrate-binding pockets that facilitate the enzyme’s preference for short branched-chain acyl-CoAs. Key residues involved in FAD binding and substrate interaction were identified, elucidating the catalytic mechanism of IVD. Additionally, we investigated the impact of various disease-associated hotspot mutations derived from different regions, demonstrating their effects on enzyme stability and activity. Notably, mutations such as A314V, S281G/F382V, and E411K resulted in substantial loss of function, while others exhibited milder effects, which is consistent with our structural analyses. These insights enhance our understanding of IVD’s enzymatic properties and provide a foundation for developing targeted therapies for IVA.

## Introduction

Isovaleryl-CoA (coenzyme A) dehydrogenase (IVD) is an intramitochondrial flavoenzyme that catalyzes the conversion of isovaleryl-CoA (3-methylbutyryl-CoA) into 3-methylcrotonyl-CoA in the leucine catabolism pathway, transferring electrons to the electron-transferring flavoprotein (ETF) [1,2]. IVD belongs to a vital family comprising diversity of acyl-CoA dehydrogenases (ACADs), which share considerable sequence and tertiary structure homology and employ a similar enzymatic mechanism for the α, β-dehydrogenation of acyl-CoA substrates [2,3]. Among these, 3 ACADs including IVD, isobutyryl-CoA dehydrogenase, and short branched-chain ACAD, are involved in amino acid catabolism, while 4 others with straight-chain substrate specificities including short, medium, long, and very long ACADs are involved in β-oxidation of fatty acids [2,4]. All of these enzymes except for very long-chain acyl-CoA dehydrogenase (VLCAD), a homodimer associated with the inner mitochondrial membrane, are soluble homotetramers with a subunit mass of approximately 43 kDa, residing in the mitochondrial matrix [5,6]. IVD is particularly crucial in the degradation of leucine, a process vital for maintaining metabolic homeostasis, fatty acid oxidation, neurological energy demands, and immune function [7].

The enzymatic reaction catalyzed by IVD is initiated by the binding of acyl-CoA substrates [8]. The mechanism of ACADs can be divided into 2 half-reactions: a reductive and an oxidative phase. In the reductive half-reaction, a catalytic glutamate residue extracts the C_α_-H hydrogen, while the C_β_-H hydrogen is transferred as a hydride to the N_5_ position of the flavin. Each subunit of IVD contains one noncovalently attached FAD molecule, imparting a characteristic yellow color to the purified enzyme [9]. The subsequent oxidative half-reaction is necessary to regenerate oxidized FAD by transferring electrons from FADH_2_ to ETF [10,11].

Deficiency in human IVD is responsible for isovaleric acidemia (IVA; OMIM #243500), a serious metabolic disorder with an autosomal recessive inheritance [12,13]. IVA is characterized by the accumulation of isovaleric acid and its derivatives, including 3-hydroxyisovaleric acid, isovalerylcarnitine, and isovalerylglycine, in cells, blood, and urine [14–17]. This metabolite accumulation leads to severe organ damage. Clinical manifestations of IVA include poor feeding, vomiting, lethargy, seizures, developmental delays, metabolic acidosis, and sweaty feet odor [18,19]. Over 60 disease-causing mutations in the IVD gene have been documented, primarily point mutations, along with splice site mutations, nonsense mutations, missense mutations, deletions, and insertions [20–24]. However, an increasing number of IVD variants with unknown functional effects are being reported across diverse ethnicities [18]. Thus, a deeper comprehension of the functional implications of IVD mutations is essential for improving diagnostic and therapeutic strategies.

Previous study has reported the crystal structure of human IVD, identifying E286 as a key catalytic residue [1,25]. However, limitations exist in the research; substrate or substrate analogs were not added during purification or crystallization. Notably, isovaleryl-CoA was modeled into the active site of IVD using density observed for CoA persulfide, but the clear tracing of isovaleryl-CoA density was lacking, resulting in an incomplete structural understanding of the IVD–substrate complex. Comprehensive structural investigations of the complete IVD–substrate complex are essential for elucidating the dynamics and catalytic mechanisms of IVD, thereby enhancing our understanding of the pathophysiology underlying IVA.

In this study, we presented the cryo-electron microscopy (cryo-EM) structures of human IVD both in its apo form and in complex with its substrates, isovaleryl-CoA and butyryl-CoA. These structures reveal a tetrameric architecture with distinct substrate-binding pocket that facilitates the enzyme’s preference for branched-chain acyl-CoAs. We have further investigated critical residues involved in the onset of IVA, which provides valuable insights into their effects on the biochemical and structural properties of IVD.

## Results

### Cryo-EM structure of IVD

**Fig. 1.**
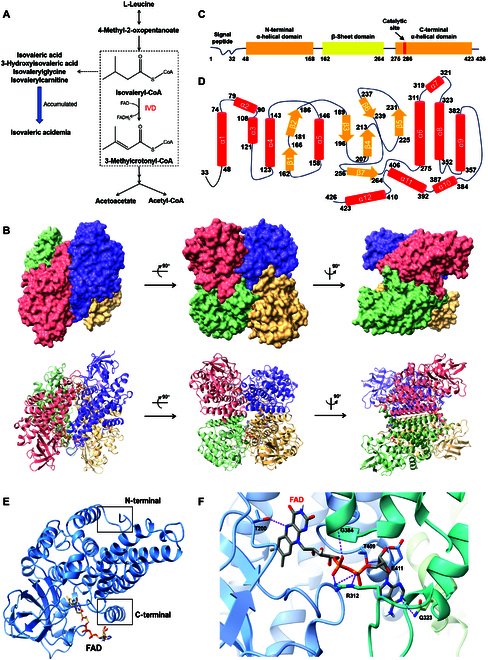
Overall cryo-EM structure of IVD combined with FAD. (A) IVD participates in the third step of catabolism of l-leucine, converting isovaleryl-CoA to 3-methylcrotonyl-CoA. Dysfunction of IVD leads to accumulation of relative isovaleric acid and derivatives, resulting in IVA. (B) Domain diagram of IVD monomer, with residues of domain boundaries and catalytic site being highlighted. (C) Schematic diagram of secondary structure of each monomer. α-Helices and β-sheets were represented with red cylinders and yellow arrows, respectively. (D) Overall surface structure and atomic model of IVD were displayed in different views. Monomers in tetramer were colored distinguishingly. (E) Focused cartoon model of IVD monomer binding with a FAD molecule. Three domains are arranged in a “U” shape, with the 2 α-helical domains in close proximity. (F) Residues involved in forming hydrogen bond interactions with FAD were pointed out around zoom-in field of cofactor binding pocket. Adjacent monomers were differentiated with colors.

As a critical enzyme in the catabolic breakdown of leucine, IVD catalyzes the conversion of isovaleryl-CoA into 3-methylcrotonyl-CoA. Dysfunction of IVD can lead to the accumulation of isovaleric acid, 3-hydroxyisovaleric acid, isovalerylglycine, and isovalerylcarnitine, ultimately resulting in IVA (Fig. 1A). Thus, cryo-EM structural analysis on IVD was conducted to provide valuable insights into the enzyme and the related disease.

The IVD sample prepared for cryo-EM was obtained through heterologous expression of human IVD in *Escherichia coli* BL21 (DE3) and characterized with size exclusion chromatography as well as sodium dodecyl sulfate–polyacrylamide gel electrophoresis (Fig. S1). During purification, the protein sample exhibited a conspicuous yellow color, indicative of its interaction with FAD, a cofactor that accepts electrons and stabilizes the conformation of IVD. Thus, the original structure of IVD was resolved in the form of only combining with FAD molecules, achieving a resolution of 2.55 Å. Consistent with other ACADs, including short-, medium-, and long-chain ACADs [6,26,27], IVD is characterized by a tetrameric architecture composed of 4 identical subunits (Fig. 1B). Each pair of monomers forms a centrally symmetric arrangement with the remaining subunits, twisting tightly together. A pocket-like space exists between adjacent monomer attachment surfaces, providing a binding site for catalytic substrates and cofactors.

Analysis of the secondary structure of IVD reveals a well-organized domain distribution, broadly categorized into 3 regions: 2 α-helical domains and 1 β-sheet domain (Fig. 1C). The 2 α-helical domains are situated at the N-terminal and C-terminal regions, while the central region comprises the β-sheet domain, which are composed of 5 α-helices, 7 β-strands, and 7 α-helices, respectively (Fig. 1D). These domains are arranged in a “U” shape, with the 2 α-helical domains in close proximity (Fig. 1E). Furthermore, the C-terminal α-helical domain and the β-sheet domain from one subunit create an exposed pocket, which, together with the C-terminal α-helical domain of the adjacent monomer, provides a structural basis for binding and catalytic functions. To enhance protein stability for cryo-EM analysis, additional FAD was incorporated into the sample buffer. Each tetramer of IVD can bind 4 FAD molecules. The base of the binding pocket, where the isoalloxazine ring of FAD resides, is predominantly composed of hydrophobic amino acids, while the opening toward the neighboring monomer accommodates the adenosine portion (Fig. 1F). The cryo-EM structure reveals that each FAD molecule is coordinated by residues from 2 adjacent monomers within the IVD tetramer. Specifically, T200 from one monomer forms hydrogen bonds with the isoalloxazine group of FAD, while Q323 from the neighboring monomer stabilizes the adenosine moiety. Other residues including R312, G384, T409, and E411, which interact with the ribose and pyrophosphate group, arise from bordering monomers as well. This inter-subunit binding mode implies that FAD not only participates in catalysis but also bridges monomers to facilitate tetramer assembly.

### Structural insights into the substrate-binding pocket of IVD

IVD is classified as a short-branched chain ACAD, exhibiting the highest activity for isovaleryl-CoA. Glutamate 286, identified as the catalytic base of IVD, is situated at the bottom of the active pocket. This glutamate functions as a proton acceptor during the dehydrogenation reaction and abstracts the α-hydrogen from substrates [1]. To elucidate the complex structure with its substrates, an IVD construct with the E286A mutation was expressed and purified for cryo-EM preparation to minimize conformational instability caused by the process of enzymatic reaction. Upon incubation with isovaleryl-CoA and butyryl-CoA, complexes of IVD with both substrates were resolved at resolutions of 3.35 and 2.91 Å, respectively.

**Fig. 2.**
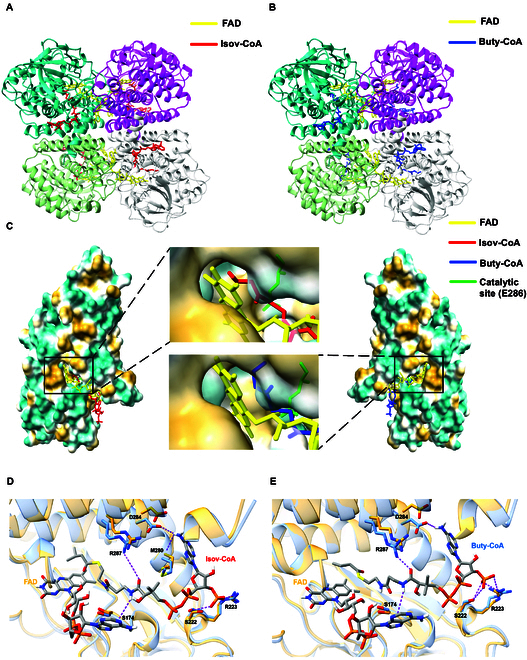
Comparison between complexes of IVD binding with isovaleryl-CoA and butyryl-CoA. (A and B) The whole IVD structure combined with isovaleryl-CoA (A) or butyryl-CoA (B) was presented in cartoon model. Two substrates and FAD were distinct with different color. (C) The molecular lipophilicity potential surfaces of IVD complexes, with cyan representing hydrophilic potential and yellow representing hydrophobic potential. Deep-in views of substrate-binding pocket were displayed in windows. (D and E) Zoomed views showing the effects of isovaleryl-CoA (D) and butyryl-CoA (E) binding on the structure of substrate pocket. Residues involved in hydrogen bonds with isovaleryl-CoA were labeled, with bonds represented by purple dashed lines.

Following the addition of the 2 acyl-CoAs, the refinement process revealed additional tubular densities in the active site that ran partially parallel to the riboflavin portion of FAD, which were well-modeled as isovaleryl- and butyryl-CoA (Fig. 2A and B). FAD enters the pocket through a space formed at the junction of neighboring monomers, while the acyl-CoAs adhere to the surface of one monomer and enter through an opening on the opposite side, allowing their fatty acyl portions to interact with the FAD ring deep in the pocket. The residues surrounding the active site create a relatively hydrophobic environment, facilitating the binding of both acyl-CoAs in a similar orientation (Fig. 2C). Comparison of the 2 acyl-CoAs in terms of binding revealed a twist alternation at the fatty acyl portion, potentially attributed to the additional side-chain methyl constraining space within the pocket.

The residues involved in binding with 2 acyl-CoAs are predominantly identical (Fig. 2D and E). D284 and M280 form hydrogen bond interactions with adenosine at N_6_, while S222 and R223 contribute to interactions with the 3′-phosphate. These interactions confirm that the CoA on the outer part of the pocket is close to the monomer, while the remaining portion extends into the pocket, riveted by S174 and R287 from opposing directions. The elongated acyl-CoAs are stabilized around the active site by participating residues, contributing to a more compact and stable structure of IVD.

### Structural basis for the substrate specificity of IVD

The ACAD family utilizes a diverse range of acyl-CoAs, differing in carbon chain length as substrates [28]. IVD differs from other ACADs in 2 primary features: the varying chain lengths and the presence of an additional branched moiety in its substrates. Although IVD can catalyze dehydrogenation of substrates ranging from C_4_ to C_6_, it exhibits the highest activity for isovaleryl-CoA, a C_4_ straight chain with a methyl group at the C_β_ position. In comparison, the activity of IVD for butyryl-CoA, which lacks a branched group, is no more than 30% of that for its optimal substrate. ACADM, another member of the ACAD family, exhibits broader substrate specificity, accommodating CoAs with straight chains ranging from C_6_ to C_12_ [29]. Architectural comparison between IVD and ACADM reveals distinguishing features that elucidate their substrate specificity.

**Fig. 3.**
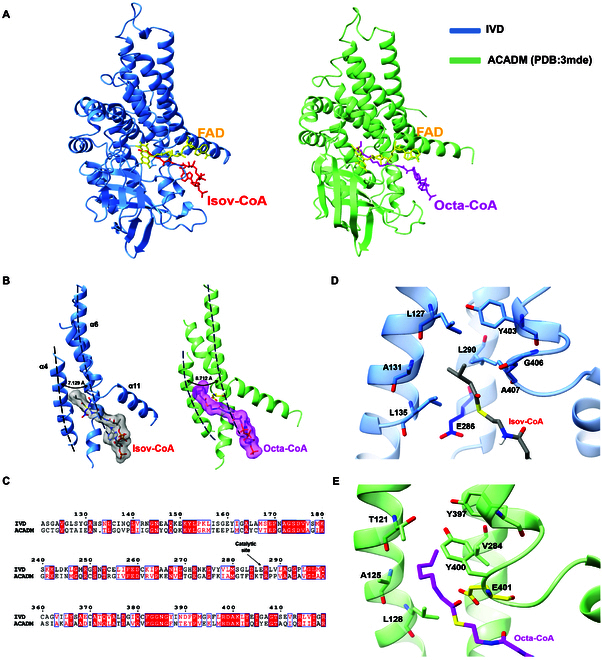
Comparison between structures of IVD and ACADM combined with their respective substrates to investigate structural basis for the substrate specificity. (A) General comparison between IVD and ACADM indicates a structural conservation in the ACAD family. Green-colored ACADM shows a relatively looser substrate pocket than that of IVD. (B) The α-helices 4, 6, and 11 that form the pocket wall were shown compared to the corresponding structure in ACADM. Around the fatty acyl portion of substrates, IVD shows a narrower passageway that prevents it from accommodating substrates with longer carbon length. (C) Sequence alignment between IVD and ACADM in residues forming substrate pocket. Conserved sequences were highlighted in red boxes. (D) Residues responsible for determination of substrate specificity were exhibited in IVD complexed with isovaleryl-CoA. L127 and L290 form a barrier at the bottom, while G406 and A407 provide space for branched chain. (E) Residues corresponding to (D) were exhibited in ACADM complexed with octanoyl-CoA. T121 and V284 provide a wider pathway to accommodate longer substrates, while G406 and A407 form the barrier to prevent the insertion of branched chain.

Although IVD and ACADM share overall structural conservation to maintain similar catalytic abilities (Fig. 3A), subtle differences in the pocket region play a crucial role in determining substrate specificity. The helices α4, α6, and α11 from different domains constitute the main portion of the catalytic pocket, with 2 parallel helices α4 and α6 forming the depth-determining bottom, while the remaining helix α11 acts as a barrier that restricts the extension of branched chain from substrates (Fig. 3B). Although residues surrounding the pocket, such as A131 and L135 (A125 and L128 in ACADM), show considerable conservation between IVD and ACADM (Fig. 3C), they exhibit shorter distances between side chains at the corresponding positions and make the pathway narrower. Notably, T121 and V284 reside at the bottom of the pocket in ACADM, while the corresponding residues in IVD are L127 and L290, which possess more compact side chains and provide wider space (Fig. 3D and E). These residues, located in the helices adjacent to the substrate side, contribute to differences in conformation, resulting in a narrower pocket in IVD to prevent it from accommodating substrates with longer carbon chains.

Differences in the horizontal helices (α11) between IVD and ACADM, as illustrated in Fig. 3B, are noted to be responsible for matching branched-chain acyl-CoAs in IVD. Indeed, conserved residues are positioned in the helix between the 2 enzymes, including Y403 and I405 (Y397 and I399 in ACADM), to ensure basic common function. G406 and A407, located at a critical loop position in the middle of the helix, provide suitable space for the insertion of branched chains from substrates (Fig. 3D). In contrast, Y400 and E401 occupy the corresponding positions in ACADM, presenting greater spatial hindrance that prevents the insertion of branched chains (Fig. 3E). Furthermore, the catalytic base of IVD, glutamate E286, is situated at the bottom of the binding pocket, nearly 120 amino acids away from the corresponding position of the ACADM catalytic base (E401). The 2 catalytic bases are separated by distinct domains, and the differences in their distance as well as angle relative to the substrate C_α_ may influence their proton-accepting capacity, thereby affecting the enzyme’s catalytic efficiency for various substrates.

### Functional roles of IVA disease-associated mutations

IVA, resulting from the dysfunction of IVD, is a rare autosomal recessive disorder. Due to the disruption of normal metabolism of leucine, the excess intermediate isovaleryl-CoA is converted to isovaleric acid, 3-hydroxyisovaleric acid, isovalerylglycine, and isovalerylcarnitine, leading to organic acidemia. Although IVA is a rare disease, patients are spread all over the world, with individuals from different regions carrying distinct key missense mutations. Based on molecular analysis of these patients from diverse areas, we constructed mutants including A314V (United States and Germany), S281G/F382V (Korea), R53P (Mexico), R53C (China), and A300V and E411K (Turkey) [13,24,30–33]. To dig into the underlying mechanisms of these mutations associated with IVA, we conducted biochemical and structural analysis on the IVD mutants.

**Fig. 4.**
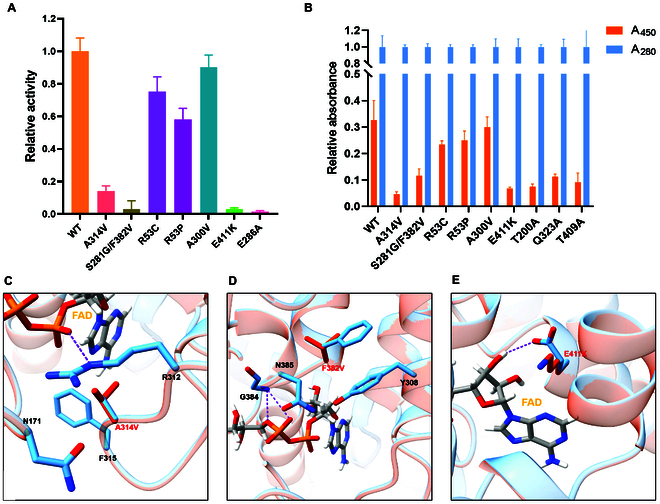
Effects of disease-associated mutations on the function and structure of IVD. (A) Activity was measured by PMS-DCPIP coupled assay as described in Materials and Methods. Isovaleryl-CoA (200 μM) was added into the mixture as substrate, and the absorbance at 600 nm in 1 min was recorded as the result to calculate enzyme activity. E286A is the mutant in catalytic site that served as a negative reference. (B) Absorbance at 450 and 280 nm of WT and mutants was detected to indicate the FAD binding capacity of IVD. When *A*_280nm_ was uniformed to ensure equivalent concentration of protein, *A*_450nm_ was regarded as a reference for comparison of FAD binding ability. T200A, Q323A, and T409A are mutants in residues having direct interactions with FAD, which act as negative reference. (C to E) Zoomed views presenting the effects of the mutations with severe decreased enzyme activity on the structure of IVD and its interaction with FAD: A314V (C), F382V (D), and E411K (E). The WT IVD structure was exhibited in cyan, while the predicted structures of mutants were colored in orange. The hydrogen bonds were shown as purple dashed lines. Every result point was averaged from repeated measurement for 3 times.

Results of size exclusion chromatography (SEC) confirmed successful expression and purification of all mutants. Under identical heterologous expression conditions, the peak protein concentrations after SEC elution were quantified as follows: wild type (WT) (6.5 mg/ml), A314V (3.2 mg/ml), S281G/F382V (2.8 mg/ml), R53P (0.9 mg/ml), R53C (0.7 mg/ml), A300V (5.7 mg/ml), and E411K (3.3 mg/ml). Notably, R53P and R53C exhibited a more than 80% reduction in yield. The melting temperature (*T*_m_) of the mutants, compared to the WT, presented varying degrees of decrease, with A300V maintaining its original level, which indicated the interference with IVD thermostability caused by mutations (Fig. S3). The results of activity assays demonstrated that A314V, S281G/F382V, and E411K led to a severe loss of function, with no more than 20% of enzyme activity remaining, while R53C, R53P, and A300V exhibited relatively milder decreases in enzyme activity (Fig. 4A). Additionally, the purified proteins displayed a distinct yellow color, attributed to their interaction with FAD. The amount of FAD bound to IVD was quantified by measuring the absorbance at 450 nm [34]. It is notable that A314V, S281G/F382V, and E411K, which exhibited a sharp decrease in enzyme activity, also demonstrated a more pronounced reduction in FAD binding capacity, whereas R53C, R53P, and A300V showed a smaller decrease (Fig. 4B).

To further elucidate the role of these mutations in IVA, we located them within the corresponding regions of IVD and examined their effects on the protein architecture. As anticipated, the mutants A314V, S281G/F382V, and E411K, which have displayed a sharp decline in enzyme activity, also play crucial roles in FAD binding according to structure analysis. A314 and F382 are located at the loop positions between helices approaching FAD from different directions, where R312 and G384 forming hydrogen bond interactions with FAD exist nearby (Fig. 4C and D). Their transition to the branched amino acid valine may have disrupted the stability of the loop structure, thereby affecting the relative positions of R312 and G384 in the structure and breaking their interaction with FAD. A314V makes greater clashes with surrounding residues including N171, F315, and R312, while F382V disturbs its interactions with N385 and Y308. Besides, E411 directly interacts with FAD through hydrogen bond, and its mutation makes a transition from negatively charged glutamate to positively charged lysine, critically disrupting this interaction. In contrast, R53, A300, and S281 are positioned far from the binding pocket and pose milder effects on the structure of IVD. These findings are consistent with the previously mentioned biochemical results.

## Discussion

IVD acts as a vital enzyme involved in the catabolic breakdown of leucine, converting isovaleryl-CoA into 3-methylcrotonyl-CoA. Previous study has elucidated the crystal structure of IVD, but their samples were purified and prepared without any addition of relevant acyl-CoA. However, the density of CoA persulfide was discovered within the binding pocket during structural refinement, ultimately determining a structural model that binds an undecided CoA. In our study, the cryo-EM structure of IVD and its complexes with isovaleryl-CoA and butyryl-CoA were clearly elucidated at resolutions of 2.55, 3.35, and 2.91 Å, respectively. Detailed cryo-EM statistics are shown in Table S2. Subsequent analyses on the detailed structures, along with further biochemical experiments, accurately demonstrate the mechanisms underlying enzymatic catalysis and substrate selectivity of IVD.

The overall structure of IVD uncovers a tetramer composed of 4 identical subunits, with each pair twisting tightly into a centrally symmetric arrangement, which is consistent with structural features observed in the ACAD family. Each monomer is organized into 3 neatly aligned domains: 2 α-helical domains positioned at the N-terminal and C-terminal regions, and 1 β-sheet domain occupying the central area. The 3 domains are arranged like a “U” shape, with partial invagination forming an opening pocket for substrate and cofactor binding. Notably, FAD serves as a cofactor in the reaction to accept electrons; when combined with the active pocket, the external portion forms hydrogen bonds with the adjacent monomer (Fig. 1F). Therefore, the binding of FAD also plays a critical role in stabilizing the protein structure, as validated by the modifications to the buffer system, where its addition reduced the protein’s propensity for precipitation. Residues T200, G384, R312, T409, E411, and Q323 participate in hydrogen bond interactions with FAD, with some mutations linked to the onset of IVA.

The complex structures of IVD with isovaleryl-CoA and butyryl-CoA were determined to investigate underlying mechanisms of catalysis. Binding experiments between WT IVD and its substrates did not yield accurate fitting results, leading us to speculate that the intense enzymatic reaction has affected the determination of binding parameters and may impact subsequent cryo-EM preparations. Thus, a mutant on the active site, E286A, was constructed and exhibited a stable combination with isovaleryl-CoA and butyryl-CoA, with apparent dissociation constant (KD_app_) of 848 and 494 nM measured by surface plasmon resonance (SPR) (Fig. S2), which was utilized for cryo-EM sampling. At the bottom of the active pocket, where the fatty acyl portion and the FAD ring reside, E286 extends its carboxyl group to approach the C_α_–C_β_ bond of the substrate to abstract proton. Comparison of the 2 acyl-CoAs in terms of binding revealed that butyryl-CoA exhibits a twist alternation in the fatty acyl portion, resulting in a longer distance and a less favorable angle between E286 and the C_α_–C_β_ bond of the substrate, which may contribute to its markedly lower catalytic efficiency compared to isovaleryl-CoA.

The ACAD family comprises various ACADs, among which IVD differs from the other ACADs in 2 primary features regarding substrate specificity: a shorter acyl chain length (C_4_ to C_6_) and a branched-chain preference for its substrates. A detailed structural comparison around the active pocket between IVD and ACADM, which accommodates acyl-CoAs with straight chains ranging from C_6_ to C_12_, reveals distinguishing characteristics in substrate specificity. L127 and L290 are located at restricted position deep within the IVD pocket, and compared to T121 and V284 at the corresponding position of ACADM, they exhibit a closer side-chain distance that narrows the pocket. When the carbon chain length of substrate increases to C_7_ or even more, this critical bottleneck will prevent longer carbon chains from extending inward, thereby conferring specificity for shorter chain substrates. On the other hand, Y400 and E401, located on the side of the ACADM pocket, are responsible for greatly limiting the degree of freedom in lateral space. In contrast, the corresponding positions in IVD are occupied by glycine and alanine, which have smaller steric hindrance, providing greater room for the accommodation of branched chains.

Deficiency of IVD leads to IVA, a rare autosomal recessive disorder, with symptoms including feeding intolerance, developmental delay, metabolic acidosis, and pancytopenia. The construction of mutants based on hotspot mutations from various regions, including A314V (United States and Germany), S281G/F382V (Korea), R53P (Mexico), R53C (China), and A300V and E411K (Turkey), was undertaken to investigate their effects on the disease. Based on purification results and biochemical experiments, these mutations can be classified into three categories regarding their impact on disease manifestation. A314V, S281G/F382V, and E411K led to a severe loss of function, with enzyme activity remaining below 20%. R53P and R53C exhibited reduced enzyme activity to a lesser extent, but showed lower expression levels during purification. A300V displayed enzyme activity and purification performance closest to the WT, with relatively mild symptoms in cases, including vomiting and tachypnea [24].

Our structural analysis of IVD mutations reveals that destabilization of FAD binding directly impairs enzymatic activity, which correlates with severe neonatal-onset IVA cases presenting with life-threatening metabolic acidosis. Conversely, mutations retaining partial activity are associated with milder phenotypes, such as episodic vomiting triggered by metabolic stress. These findings highlight the importance of FAD-dependent structural integrity for IVD function and suggest that pharmacological chaperones targeting cofactor binding could mitigate enzyme instability in specific mutations. Furthermore, the distinct substrate-binding geometry of IVD, particularly the G406/A407 motif, provides a structural blueprint for designing substrate analogs to competitively inhibit toxic metabolite accumulation in IVA patients.

In summary, our study provides comprehensive insights into the structural and functional characteristics of IVD, highlighting its critical role in leucine metabolism and the implications of specific mutations associated with IVA. Through cryo-EM analysis, we elucidated the enzyme’s tetrameric structure, substrate-binding mechanisms, and the importance of FAD in stabilizing the protein. The comparative analysis of IVD and other ACADs revealed distinct features that govern substrate specificity, particularly the preference for short and branched-chain acyl-CoAs. Furthermore, our investigation into disease-associated mutations underscores the functional consequences of these alterations on enzyme activity and stability, contributing to the understanding of the molecular basis of IVA. These findings not only enhance our understanding of IVD’s enzymatic properties but also pave the way for potential therapeutic strategies targeting this rare metabolic disorder.

## Materials and Methods

### Cloning, expression, and purification of IVD

The gene coding *IVD* was chemically synthesized based on the codon-optimization cDNA sequence of human *IVD*. The fragment encoding residues 33 to 426 dragging an 8×His tag at the N terminus was cloned into the pET-28a vector (GenScript). Mutants were constructed based on the WT plasmid by polymerase chain reaction using 2× Phanta Flash Master Mix (Vazyme). The constructed plasmid was transformed into the *E. coli* BL21 (DE3) grown in LB medium with 0.05 mg/ml kanamycin. When the bacteria proliferated to an OD_600nm_ ranging from 0.6 to 0.8 cultured at 37 °C, 500 μM isopropyl-β-d-thiogalactopyranoside was added to induce cultivation for 16 h at 16 °C. Cells were harvested by centrifugation at 4,000 rpm and resuspended with lysis buffer containing 50 mM tris-HCl, pH 7.4, 500 mM NaCl, and 5% glycerol. Collected cells were then lysed with high-pressure machine and centrifugated at 12,000 rpm for separating cell debris from proteins. The supernatant was prepared for subsequent purification with affinity chromatography using Ni-NTA (Qianchun Bio) beads followed by size exclusion chromatography using a Superdex 200 10/300 GL column (GE Healthcare). The finally purified protein was saved in the SEC buffer containing 50 mM tris-HCl, pH 7.4, 150 mM NaCl, and 20 mΜ FAD.

### Cryo-EM sample preparation

Purified proteins from size exclusion chromatography were used for cryo-EM analysis. The complex sample is obtained by incubating protein with substrates at 37 °C for 15 min. Each sample (3 μl) was applied onto an R 1.2/1.3 holey carbon film Au grids and then blotted for 5 s with a blot force of 2. After blotting, samples were plunge frozen by liquid ethane and stored for cryo-EM data collection.

### Cryo-EM data collection and processing

Data collection was conducted at a 300-kV Titan Krios electron microscope (Thermo Fisher Scientific). Raw images were recorded by a K3 electron detector with GIF BioContinuum HD energy filter (Gatan) at a magnification of 105,000×, which equals a physical pixel size of 0.824 Å [35]. Initial image collections were delt with motion correction using MotionCor2. The CTFFIND4 in CryoSPARC V4.3.1 [36] was used to estimate the contrast transfer function. Subsequently, particles were picked and sorted with 2-dimensional (2D) classification to be organized by structure features. These particles were then grouped by ab initio classification to form primary 3D models, based on which refinement was conducted to filter heterogeneous structures and generate refined 3D models. Final maps were obtained from nonuniform refinement. Model building and further refinement was preformed using Coot V0.9.6 [37]. Finally, constructed models were refined in real space carried by PHENIX V1.17.1 [38].

### Enzyme activity assay

The activity assay of IVD was carried out by the method of phenazine methosulfate/2,6-dichlorophenolindophenol (PMS/DCPIP) coupled color reaction, as described previously [39]. Phenazine methosulfate and 2,6-dichloroindophenol serve as substitute intermediate and terminal electron acceptor, with reduced DCPIP reacting as blue color and being measured spectrophotometrically following the decreased absorbance at 600 nm. A standard assay mixture is composed of 20 mM phosphate buffer, pH 7.4, 1.5 mM PMS, 100 μM DCPIP, 30 μM EDTA, and gradient concentrations of corresponding acyl-CoAs. Diluted protein (10 μg) was added to start the reaction with a final volume of 200 μl. The reaction mixture was incubated at 37 °C using a 96-well plate for 3 min, and *A*_600nm_ was timely recorded every 30 s. Output absorbance values were used to calculate velocity of enzymatic reactions and derive kinetic parameters by fitting into the Michaelis–Menten equation. All the values were obtained from 3 repeated measurements.

### Thermostability assay

WT and all mutants were diluted into a concentration of 1 mg/ml before measurement except for R53C and R53P with a lower concentration from purification. An appropriate amount of sample was inhaled into the capillaries and loaded onto the Prometheus NT.48 instrument (NanoTemper Technologies) with sequential arrangement. A linear ramp of 1 °C per min was set at a range of 35 to 85 °C, and the fluorescence variations at 300 and 350 nm were measured. Data were processed to draw the thermal stability curve and calculate the *T*_m_ values. Every point represents the averages from 3 repeated measurements with standard errors.

## Data Availability

Electron density maps and atomic coordinates of structures in this study have been deposited as follows: IVD complex with FAD (PDB: 9JQ3; EMDB: EMD-61721), IVD complexed with isovaleryl-CoA (PDB: 9JQ5; EMDB: EMD-61723), and IVD complexed with butyryl-CoA (PDB: 9JQ4; EMDB: EMD-61722). All other data are provided in the manuscript and Supplementary Materials.
