# Supplementary material for: Structural Insights into Isovaleryl-Coenzyme A Dehydrogenase: Mechanisms of Substrate Specificity and Implications of Isovaleric Acidemia-Associated Mutations
Source: Research (Wash D C). 2025 May 28;8:0661. doi: 10.34133/research.0661 (PMC12369846; doi:10.34133/research.0661)
Supplement: Supplementary 1 — Figs. S1 to S4 Tables S1 and S2 [file research.0661.f1.pdf]

Supplementary Materials

Figs. S1 to S4
Tables S1 to S2

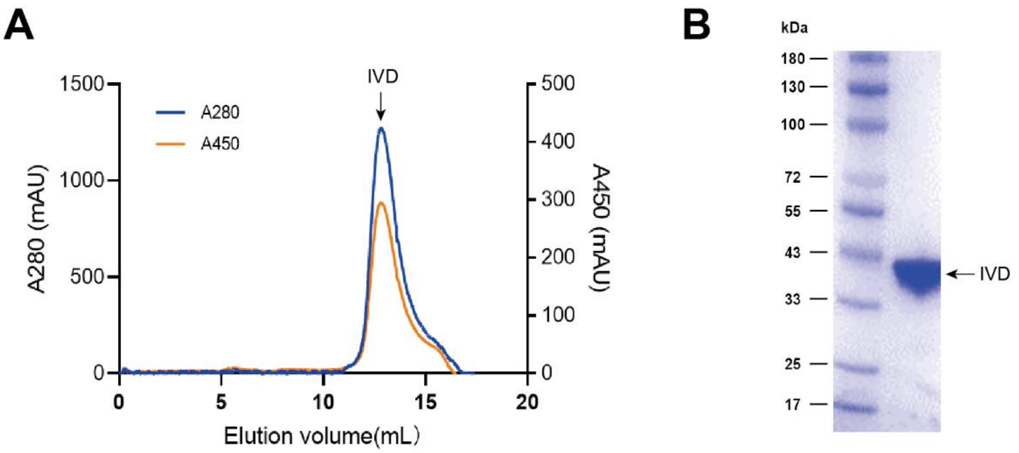

Fig. S1. Biochemical characterization of human IVD. (A) Size exclusion chromatography curve of IVD. The absorbance at 280 nm and 450 nm was recorded. (B) SDS-PAGE result of purified IVD. The obtained sample with a high purity exhibited as a monomer at around 42 kDa.

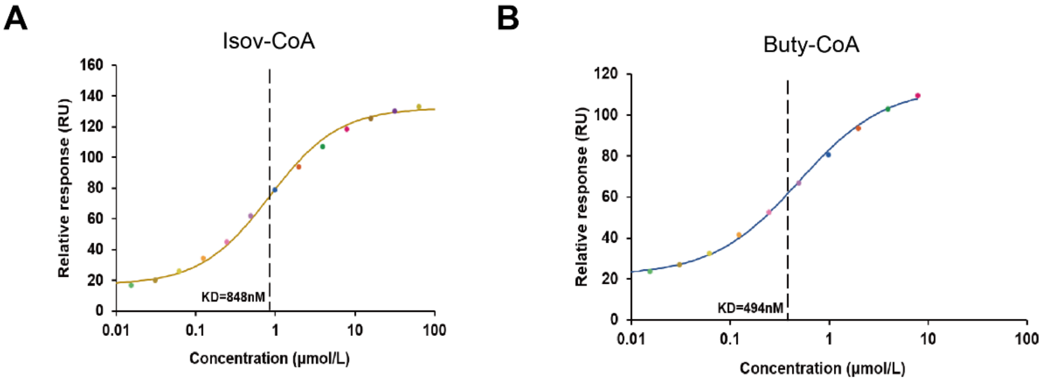

Fig. S2. Affinity determination of IVD-E286A for isovaleryl-CoA and butyryl-CoA using surface plasmon resonance (SPR). The results indicated that IVD with mutation E286A showed a high affinity for both isovaleryl-CoA (A) and butyryl-CoA (B), with KD_{app} of 848 nM and 494 nM, respectively.

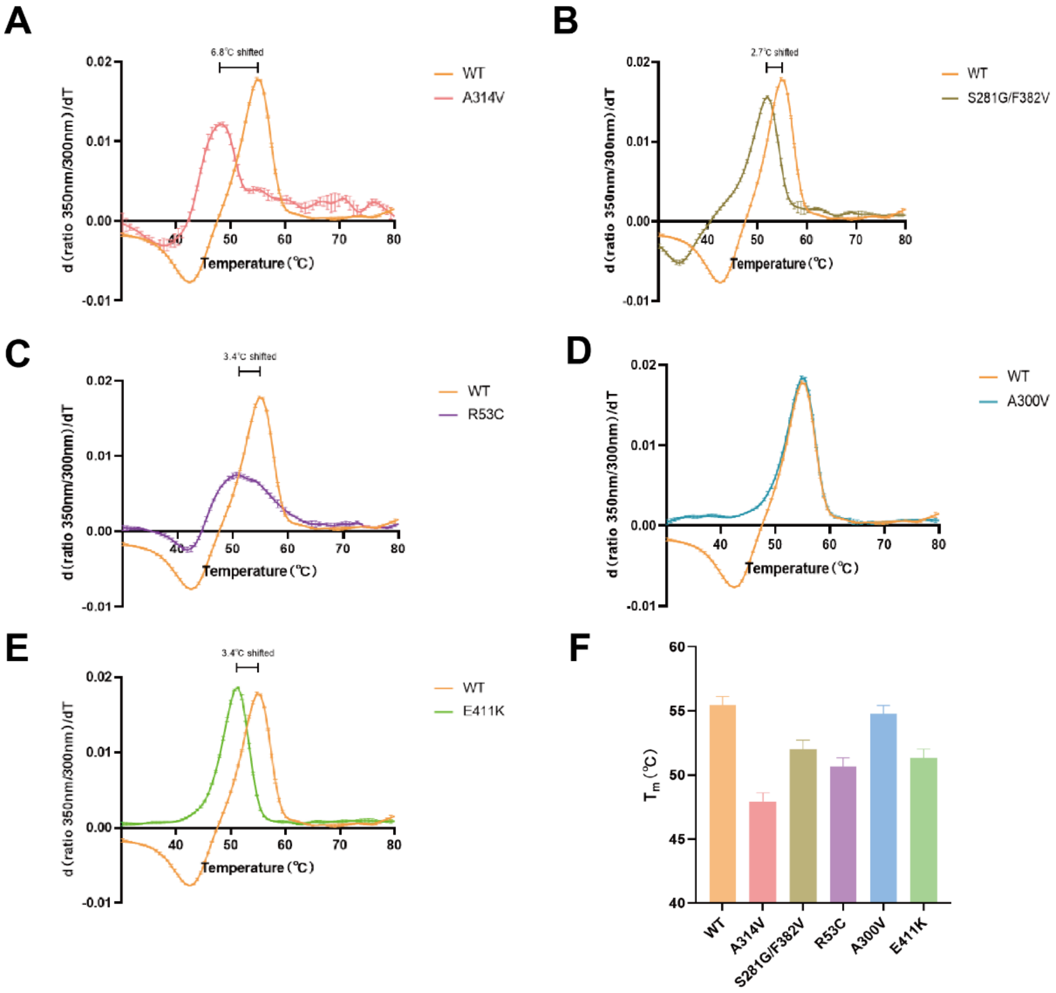

Fig. S3. Effects of disease-associated mutations on thermostabilities of IVD. The first derivates of fluorescence intensity of 350/300 nm ratio against temperature were plotted to describe the thermostabilities of A314V (A), S281G/F382V (B), R53C (C), A300V (D), E411K (E) compared with WT. The abscissa corresponding to the peak is the T_m value, which was displayed in comparison (F).

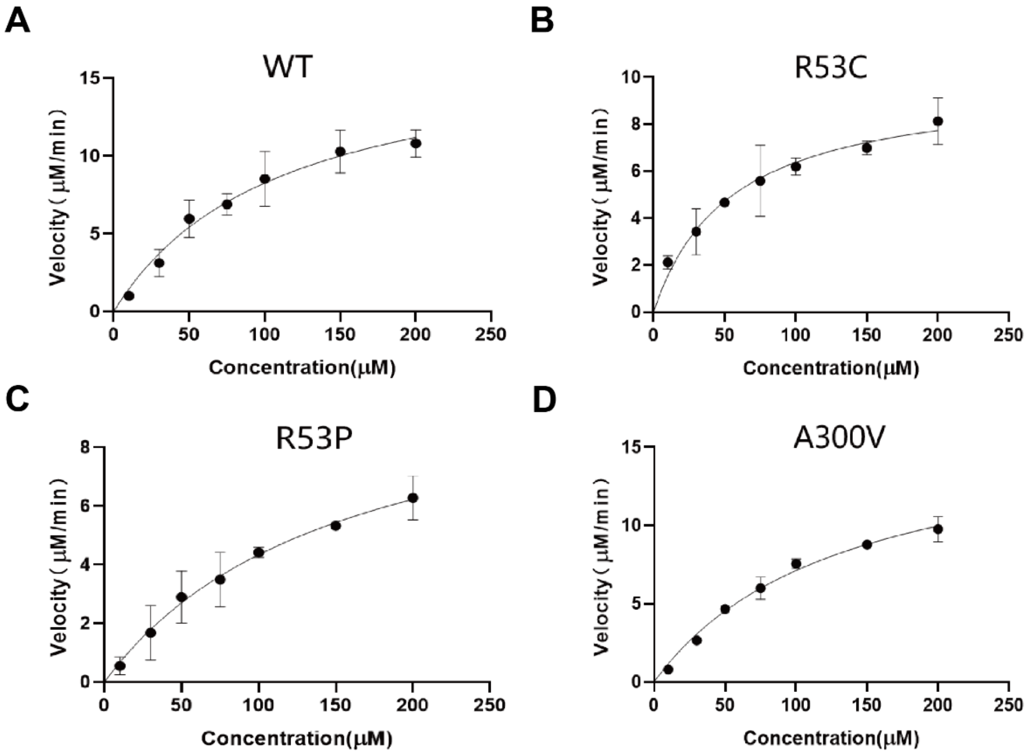

Fig. S4. Impact of disease-associated mutations on kinetic properties of IVD. The curve was fitted into the Michaelis-Menten equation based on data from enzyme activity assays. A standard assay mixture is composed of 20 mM phosphate buffer, pH 7.4, 1.5 mM PMS, 100 μM DCPIP, 30 μM EDTA and gradient concentrations of corresponding acyl-CoAs. 10 μg of diluted protein was added to start the reaction with a final volume of 200 μL. Mutations with severe decrease on activity cannot be fitted into the equation due to a very low activity. Others with relative milder decrease including R53C (B), R53P (C) and A300V (D) were recorded compared to the WT (A).

Table S1. Kinetic parameters of WT IVD and disease-associated mutants.

Mutations	K_m (μM)	V_{max} (μM/min)
WT	109.3	17.31
A314V	N.D.	N.D.
S281G/F382V	N.D.	N.D.
R53C	54.34	9.834
R53P	154.2	11.03
A300V	132.9	16.59
E411K	N.D.	N.D.

Activity was measured by PMS-DCPIP coupled assay as described in methods. A standard assay mixture is composed of 20 mM phosphate buffer, pH 7.4, 1.5 mM PMS, 100 μM DCPIP, 30 μM EDTA and gradient concentrations of corresponding acyl-CoAs. 10 μg of diluted protein was added to start the reaction with a final volume of 200 μL. The values of kinetic parameters were calculated from the saturation curve of the averages of the triplicate measurements with the fitting errors.

Table. S2. Cryo-EM data collection, processing, model refinement and validation parameters.

	IVD-FAD	IVD-butyryl-CoA	IVD-Isovaleryl-CoA
**Data collection and processing**			
Detector	K3	K3	K3
Magnification	165,000	165,000	165,000
Voltage (kV)	300	300	300
Electron exposure (e /Å^2)	50	50	50
Defocus range (μm)	-1.0~-3.0	-1.0~-3.0	-1.0~-3.0
Pixel size (Å)	0.824	0.824	0.824
Symmetry imposed	C1	C1	C1
Initial particle projections (no.)	2970120	896273	1001234
Final particle projections (no.)	733147	141942	102113
Map resolution (Å)	2.55	2.91	3.35
Map resolution range (Å)	2.50~3.50	2.50~3.50	2.50~3.50
FSC threshold	0.143	0.143	0.143
**Model Refinement**			
Refinement package	PHENIX-1.17.1-3660	PHENIX-1.17.1-3660	PHENIX-1.17.1-3660
Real or reciprocal space	Real space	Real space	Real space
Model-Map CC (mask)	0.83	0.68	0.49
Model resolution (Å)	2.66	3.46	4.32
FSC threshold	0.5	0.5	0.5
B factors (Å^2, min/max/mean value)			
Protein residues	2.00/41.71/11.66	2.00/41.71/11.68	2.00/41.71/11.68
Ligand	20.00/20.00/20.00	20.00/20.00/20.00	20.00/20.00/20.00
**Model composition**			
Non-hydrogen atoms	12,052	12,248	12,252
Protein residues	1,548	1,548	1,548
R.m.s. deviations			
Bond lengths (Å)	0.005	0.011	0.006
Bond angles (°)	1.057	1.389	1.184
**Validation**			
MolProbity score	1.32	1.66	1.67
Clashscore	5.91	10.94	11.14
Rotamer outliers (%)	0.65	1.38	1.38
Ramachandran plot			
Favored (%)	98.96	98.77	98.77
Allowed (%)	0.97	1.17	1.17
Disallowed (%)	0.06	0.06	0.06
**Data availability**			
EMDB entry	EMD-61721	EMD-61722	EMD-61723
PDB entry	9JQ3	9JQ4	9JQ5
